# Identifying Controlling Nodes in Neuronal Networks in Different Scales

**DOI:** 10.1371/journal.pone.0041375

**Published:** 2012-07-27

**Authors:** Yang Tang, Huijun Gao, Wei Zou, Jürgen Kurths

**Affiliations:** 1 Research Institute of Intelligent Control and Systems, Harbin Institute of Technology, Harbin, China; 2 Institute of Physics, Humboldt University Berlin, Berlin, Germany; 3 Potsdam Institute for Climate Impact Research, Telegraphenberg, Potsdam, Germany; 4 School of Mathematics and Statistics, Huazhong University of Science and Technology, Wuhan, China; 5 Institute for Complex systems and Mathematical Biology, University of Aberdeen, Aberdeen, United Kindom; Indiana University, United States of America

## Abstract

Recent studies have detected hubs in neuronal networks using degree, betweenness centrality, motif and synchronization and revealed the importance of hubs in their structural and functional roles. In addition, the analysis of complex networks in different scales are widely used in physics community. This can provide detailed insights into the intrinsic properties of networks. In this study, we focus on the identification of controlling regions in cortical networks of cats’ brain in microscopic, mesoscopic and macroscopic scales, based on single-objective evolutionary computation methods. The problem is investigated by considering two measures of controllability separately. The impact of the number of driver nodes on controllability is revealed and the properties of controlling nodes are shown in a statistical way. Our results show that the statistical properties of the controlling nodes display a concave or convex shape with an increase of the allowed number of controlling nodes, revealing a transition in choosing driver nodes from the areas with a large degree to the areas with a low degree. Interestingly, the community Auditory in cats’ brain, which has sparse connections with other communities, plays an important role in controlling the neuronal networks.

## Introduction

Synchronization is widely observed in many fields such as coupled nonlinear systems and complex networks [1]–[6]. Especially, synchronization of distributed brain activity has been found to play an important role in neural information processing [7]–[11]. The experimentally observed brain activity, characterized by synchronization phenomena over a wide range of spatial and temporal scales, reflects the relevance for cognitive dysfunctions and pathophysiology [8]. Structurally, the analysis of the anatomical connectivity of the mammalian cortex has uncovered that large-scale neuronal networks display both high clustering and short pathlength [12], [13]. The cortical network also shows a hierarchy of complex connectivity [12], [14]–[17].

Extensive information in mammalian cortex, such as the brains of macaque monkeys and cats, has been collected [18]–[22]. Recently, hub regions, which are believed to play pivotal roles in the coordination of information flow in brain networks [22]–[24], have been identified using modern tools from complex networks [20], [22]. The hub regions of cortical networks are analyzed using node degree, structural motif, path length and clustering coefficient distributions [22]. The results in [20] highlight the influence of the topological connectivity in the formation of synchronization, revealing a few cortical areas forming a Rich-Club connectivity pattern.

Control of complex networks is a hot topic, which is closely related to synchronization of complex networks [25]–[27]. Some vertices in complex networks serve as reference sites, leaders or pacemakers [28] and drive all the other vertices toward desired targets or evolutions and thus synchronization is achieved. It is valuable to study the controllability of complex networks, especially for cortical networks due to the technical [29], [30] and neuroscience backgrounds [8], [16], [20]. By fully utilizing the structure of the networks, Lu et al. [27] found out the minimum number of controllers for the pinning synchronization control of complex network with general topology and derived some efficient criteria to judge the success of the designed pinning controllers, which are illustrated by small-world and scale-free networks to be valid and efficient for large-scale networks.

Recently, controllability of complex networks has been studied using control theory or master stability function (MSF) [25], [29], [31]. Most recently, in [32], the authors reported on a generic procedure to steer a network’s dynamics towards a given desired evolution, where techniques from MSF were used in connection with a greedy algorithm to determine a specific, suboptimal, sequence of nodes to be driven in order to control a network toward a desired dynamics. It is shown that there is a striking correlation between the suboptimal ranking and the inverse of the degree sequence [32]. However, it is still not clear how to determine the locations of optimal driving sequences, which is crucial in to achieve the most efficient controlling performance.

Understanding a complex network’s structure is beneficial to understanding its function [33], [34]. The past decade has witnessed an increasing of methods developed in this cross-disciplinary of physics community [35]. Structural properties in complex networks exist on both the microscopic level, arising from differences between single node properties, and the mesoscopic level resulting from features shared by groups of nodes. In [34], it is shown by benchmark problems how multiscale generative probabilistic exponential random graph models combined with efficient inference techniques can be used to achieve this separation of scales, resulting in an improved detection accuracy of latent classes. In [20], [36], extensive numerical evidences are given to confirm the original claims that the microscopic and mesoscopic dynamics of synchronized patterns indeed follow different routes. In [33], [37], mesoscopic analysis of networks is applied to exploratory analysis and data clustering.

In this study, we use the cortico-cortical network of cats’ brain, which is a weighted and directed network with community structure [22]. We aim to identify controlling regions (driver nodes) of brain networks of a cat, which is equivalent to enhancing controllability of cortical networks. By converting the problem of identification of controlling nodes into a single-objective optimization problem, a recent well-studied evolutionary computation method, the self-adaptive differential evolution (JaDE), is utilized to uncover the controlling nodes of the neuronal network. By utilizing JaDE, the controlling nodes are identified in microscopic, mesoscopic and macroscopic ways. In addition, the controlling nodes selected by JaDE are compared with the usual hubs [22], which are identified using node degree, betweenness centrality, closeness and node importance. In contrast to the usual hubs, most of the controlling nodes are selected from the nodes with a small degree. Our results reveal that the number of driver nodes plays a key role in the controllability of neuronal networks.

## Results

Firstly, several examples are provided to verify the performance of JaDE [38]. JaDE is used to detect the controlling nodes/areas/regions of the cortical network of cats’ brain in microscopic, mesoscopic and macroscopic ways, respectively.

We will analyze three different scales of controlling nodes/areas/regions in the cortical network: (1) the microscopic scale refers to the mean degree, the mean betweenness centrality (BC) and the mean closeness of driver nodes that are calculated under different numbers 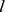 of driver nodes; (2) the mesoscopic scale corresponds to the controlling communities; (3) the macroscopic scale is the controlling nodes sorted according to their total times of serving as driver nodes.

In the following, the reliability of evolutionary computation methods is shown in terms of the convergence speed, the mean value and the best value of ten runs. In order to show that JaDE is suitable for identification of controlling nodes of the cortical network, we compare it with some well-known efficient evolutionary computation approaches CLPSO [39], jDE [40], SaDE [41] and CoDE [42]. Also, JaDE is compared with some methods in complex networks theory.

### Parameter Setting

The population sizes 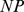 of all DEs and Particle Swarm Optimizations (PSOs) are set as 20 and the search range in each dimension is set to 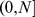 (see Materials and Methods). The maximum fitness evaluation 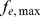 is set as 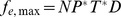, where 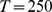 is a constant and 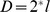 is the size of problem dimension. If a large 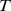 is given, the accuracy of the solutions might be refined and the computation consumption is increased linearly and vice visa. Evolutionary computation algorithms will be repeated 10 times independently for eliminating random discrepancy. Algorithms will be terminated when they achieve 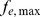.

### Comparison of JaDE with Evolutionary Computation Methods

The best value 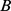 and the mean value 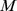 of the solutions in ten runs are listed in Table 1. The number of driver nodes is increased from 6 to 48 with a stepsize 6. 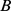 is used to describe the best solution of algorithms found in 10 times and 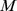 is used to represent the mean value of solutions in 10 times. Note that both the best value and the mean value of solutions are of great significance for measuring the reliability of algorithms, hence we use [43]


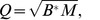
(1)

where both 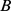 and 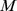 are involved. Obviously, 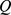 should be made as small as possible. Therefore, we also sort 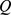 of five algorithms in an ascending way under different 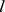 and their orders 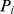. The mean order of each algorithm is calculated as follows


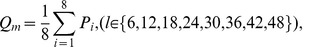
(2)

and is also listed in Table 1. Based on the mean order 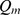, the final rank of five algorithms is obtained in Table 1 (See “Score”).

**Table 1:** Comparison among five algorithms for different 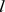 of driver nodes of the cortical network with size 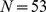, see Fig. 1.

		CLPSO	jDE	SaDE	CoDE	JaDE
	Mean	29.062	28.1897	28.0292	28.0522	28.2284
*l* = 6	Best	28.0472	27.9476	27.9219	27.9043	27.9205
	*Q*	28.5501	28.0684	27.9755	27.9782	28.074
	order	5	3	1	2	4
	Mean	14.3663	14.6584	13.8698	13.6785	13.9635
*l* = 12	Best	13.6478	13.9661	13.4678	13.4074	13.5064
	*Q*	14.0025	13.9648	13.6673	13.5423	13.733
	order	5	4	2	1	3
	Mean	9.0286	9.1186	9.1235	8.6427	8.8656
*l* = 18	Best	8.7532	8.8488	8.7087	8.4847	8.5209
	*Q*	8.8898	8.8572	8.9137	8.5634	8.6916
	order	4	3	5	1	2
	Mean	6.4847	6.283	6.5348	6.2908	6.1799
*l* = 24	Best	6.2396	6.1598	6.2228	6.0923	6.0876
	*Q*	6.361	6.1699	6.3769	6.1908	6.1336
	order	4	2	5	3	1
	Mean	5.4265	4.7714	5.1642	4.9089	4.7174
*l* = 30	Best	4.9943	4.6826	5.041	4.6569	4.675
	*Q*	5.2059	4.7	5.1023	4.7812	4.6961
	order	5	2	4	3	1
	Mean	4.8641	3.8225	4.2491	3.9336	3.8081
*l* = 36	Best	4.3501	3.7998	4.0914	3.7856	3.7968
	*Q*	4.5999	3.8039	4.1695	3.8589	3.8025
	order	5	2	4	3	1
	Mean	4.2617	3.0524	3.5074	3.084	3.0436
*l* = 42	Best	3.8142	3.0412	3.3905	3.0244	3.0324
	*Q*	4.0318	3.0424	3.4485	3.0541	3.038
	order	5	2	4	3	1
	Mean	4.13	2.3967	2.9995	2.4638	2.4119
*l* = 48	Best	3.8012	2.3825	2.8596	2.3702	2.3826
	*Q*	3.9622	2.3972	2.9287	2.4166	2.3972
	order	5	1	4	3	1
	*Q_m_*	4.75	2.375	3.625	2.375	1.75
	Score	5	2	4	2	1

The measurements of 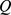 and 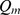 are provided in (1) and (2). “Order” is obtained by sorting 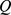 and “Score” is obtained by sorting 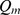 in an ascending way.

**Figure 1:**
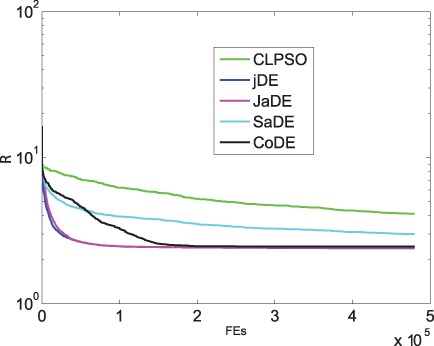
Performance of five evolutionary computation algorithms for controllability of the cortical network with different numbers 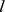 of driver nodes, when minimizing 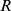 according to (8). The “FEs” here means the numbers of fitness evaluations of objective (8) or (9), when 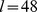.

Table 1 and Fig. 1 show that JaDE, CoDE and jDE perform better than the other two algorithms in terms of both search speed and convergence rate. From Table 1, JaDE ranks first and has good reliability of finding potential optimum with a satisfactory convergence speed. It is worth mentioning that JaDE is equipped with an elitism approach. Therefore, JaDE is able to find the global optimum when 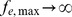. In reality, it is unreasonable to run an algorithm with infinite generations. However, the performance of JaDE is confirmed by our simulation results (Table 1 and Fig. 1). Furthermore, a series of scientific experiments in [38] reveal that JaDE is a powerful and efficient algorithm for handling real-world optimization problems. In the following, JaDE is adopted to all the following simulations.

### Comparison of JaDE with Network-based Methods

JaDE is compared with some other schemes (See Materials and Methods) from complex networks in terms of enhancement of controllability of the cortical network. The best solutions in 10 runs of JaDE under different 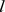 are used to produce the following results. It is worth pointing out that one can run JaDE for one time due to its reliability, as confirmed above.

**Figure 2:**
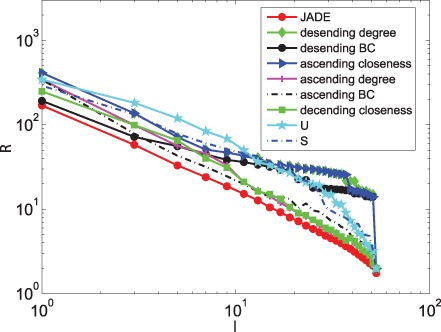
Optimizing 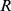 with different pinning schemes under different 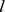.

**Figure 3:**
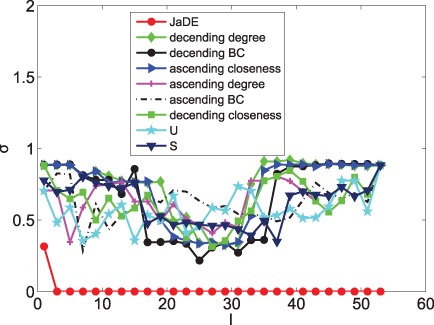
Optimizing 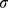 with different pinning schemes under different 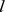.

Figs. 2 and 3 show that JaDE always performs better than the other methods. When 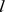 is large, the degree descending strategy, the BC descending strategy and the closeness ascending strategy are getting worse. Conversely, the degree ascending strategy, the BC ascending strategy and the closeness descending strategy are becoming better. The 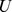 and 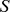-based strategies are intermediate among all the algorithms.

When only minimizing 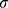 and neglecting the effect of 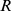, Fig. 3 shows that 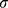 (See Materials and Methods) can easily reach zero when applying JaDE, implying that it is easy to enhance controllability in the cortical network in terms of 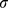. This phenomenon supports the finding in [44], [45], in which the imaginary part of the eigenvalues of network connection matrix can be neglected to measuring synchronizability of complex networks. When minimizing 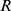 and increasing 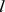, the controllability of the cortical network is becoming better using all the methods. However, when minimizing 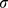 and increasing 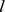, the controllability of the cortical network is getting better when only using JaDE, which is strongly different from the case of only minimizing 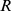.

### Controllability of the Cortical Network - a Microscopic Way

**Figure 4:**
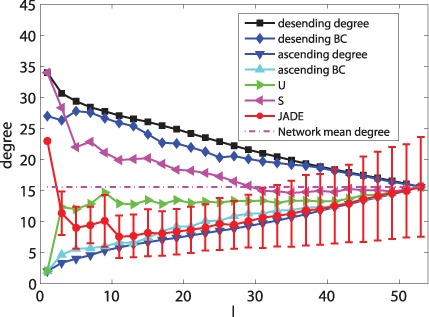
The mean values of degree information of driver nodes with various 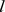 under different schemes when minimizing 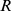.

**Figure 5:**
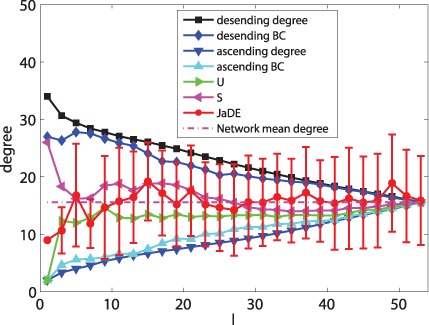
The mean values of degree information of driver nodes with various 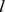 under different schemes when minimizing 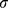.

**Figure 6:**
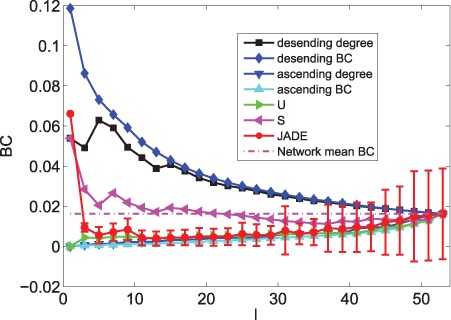
The mean values of BC information of driver nodes with various 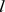 under different schemes when minimizing 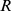.

**Figure 7:**
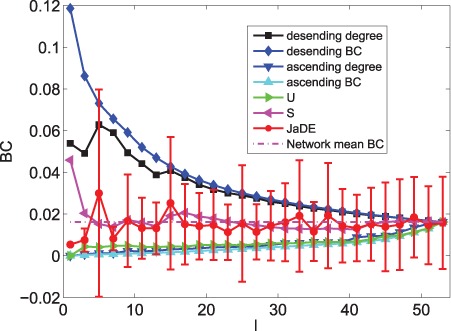
The mean values of BC information of driver nodes with various 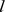 under different schemes when minimizing 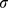.

**Figure 8:**
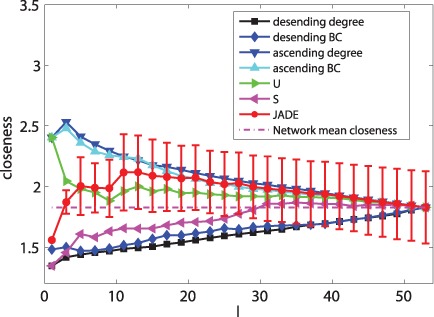
The mean values of closeness information of driver nodes with various 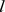 under different schemes when minimizing 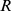.

**Figure 9:**
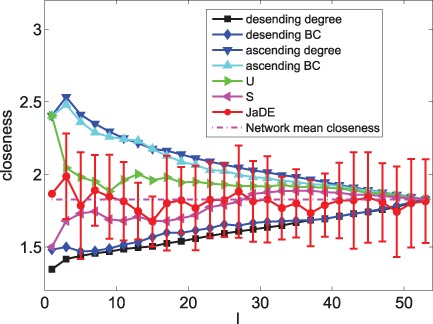
The mean values of closeness information of driver nodes with various 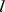 under different schemes when minimizing 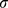.

When only minimizing 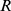, Figs. 4, 5, 6, 7, 8 and 9 depicts the mean values of degree, BC and closeness of driver nodes by various methods. Figs. 4, 6 and 8 show that, the driver nodes selected by JaDE are the nodes with a large degree, a small closeness and a large BC at the very beginning. Then, the driver nodes selected by JaDE abruptly change to the nodes with a small degree, a small BC and a large closeness, when increasing 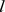. Specially, when 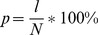 is near 20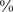, the mean value of degree of the controlling nodes selected by JaDE achieves its minimum value. After the mean value of degree of driver nodes reaches its minimum value, it increases gradually and finally attains the mean value of degree of the cortical network. As a whole, Fig. 4 shows that the mean values of degree of driver nodes display a concave shape as a function of 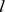. The standard deviation also becomes gradually larger when increasing 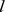. The observed phenomenon indicates that, when 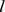 is not large, driver nodes are usually selected from the nodes with a small degree and nearly no nodes with a large degree are chosen. Some similar phenomena are observed when the BC and closeness of the driver nodes are shown (Figs. 6 and 8). This finding is consistent with the work in [29], in which the nodes with a large degree should be avoided choosing as driver nodes. It is worth mentioning that there exists a major difference with the finding in [29], i. e., when 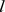 is very small, the nodes with a large degree should be considered as driver nodes, as illustrated in Fig. 4.

Different from optimizing 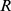, when minimizing 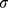, Figs. 5, 7 and 9 show that the mean values (degree, BC and closeness) of driver nodes selected by JaDE fluctuate around the mean values (degree, BC and closeness) of the network. The standard deviations (degree, BC and closeness) of driver nodes selected by JaDE keep stable when 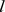 increases. All the findings indicate that one should select the nodes to make the mean values (degree, closeness and BC) of driver nodes around those of the network.

**Figure 10:**
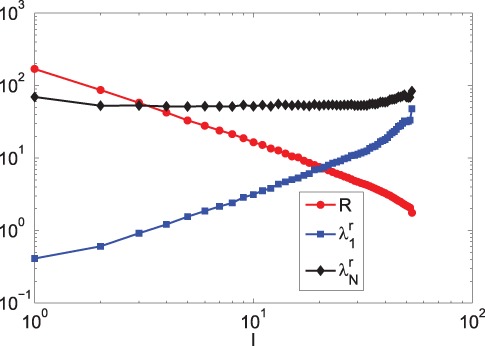
The relationship between 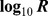, 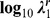, 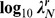 and 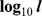 by JaDE.

Finally, the relationship between 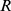, 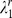, 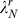 and 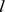 (See Materials and Methods) is investigated in terms of minimizing 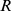. Fig. 10 shows that 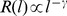, which can help to predict 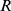 when knowing 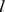. Moreover, in order to minimize 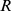 under a small 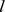, 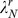 should be suppressed near a constant value and 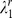 should be enlarged as much as possible. As 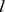 increases, both 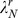 and 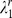 grow exponentially and the growth of the amplitude of 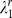 is larger than that of 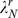. Fig. 10 illustrates that the shape of 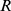 largely depends on 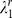. The observed phenomena indicate that 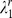 plays a more important role in minimizing 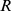 than 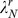 does. When 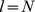, it is shown that 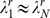, which makes 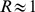. In summary, when minimizing 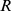, enlarging 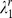 is more important than reducing 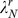. This finding is similar to our finding in [43], where only undirected complex networks are studied.

### Controlling Nodes of the Cortical Network - a Macroscopic Way

By means of JaDE, we control the cortical network under different 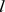 in terms of minimizing 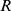 and 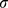, respectively. Denote


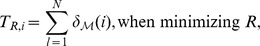
(3)

**Figure 11:**
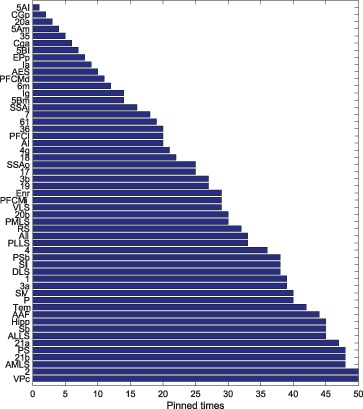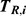 of each node in cortical networks of cat.

**Figure 12:**
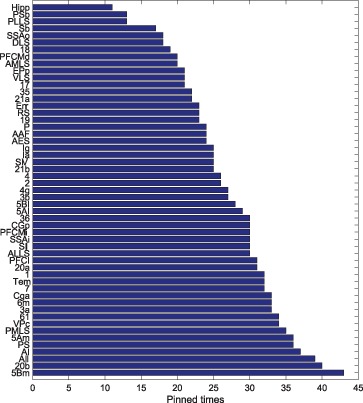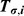 of each node in cortical networks of cat.

And


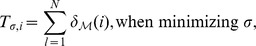
(4)

where 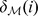 can be referred to Materials and Methods. 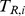 and 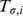 indicate the times of each node selected as driver nodes in terms of minimizing 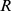 and 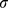, respectively. The nodes with large 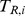 and 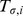 play a vital role in controlling the cortical network. The controlling nodes of the cortical network are identified for 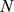 different times. Then, 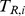 and 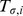 are sorted in a descending way. The results are shown in Figs. 11, 12 and Table 2. Fig. 11 shows that when minimizing 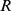, the standard deviation of 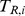 is large, which means that some nodes in the cortical network, such as VPc, 2 and AMLS, are of great importance to be controlled. Some areas are negligible to be selected as driver nodes, such as 20a, CGp and 5AI. When minimizing 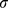, the standard deviation of 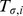 is small and nearly all the areas in the cortical network are important for minimizing 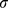. Hence, the controlling nodes are different from the usual hubs, which are generally selected from nodes with a large degree [22]. In addition, the controlling nodes in the case of minimizing 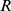 are different from those in the case of minimizing 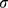 (Table 2). In order to show what factors have impacts on selection of controlling nodes, 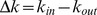 of each area in the cortical network is depicted in Table 2, where 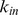 and 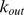 can be referred to Materials and Methods. Table 2 shows that, when optimizing 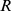, most of the controlling nodes are selected from the nodes with a large 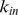 and a small 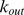. Therefore, the areas with 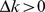 should be considered as controlling nodes when minimizing 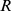. Whereas the situation is more complicated, when minimizing 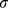 and there exist other factors which influence the selection of controlling nodes.

**Table 2:** Controlling times, Δ*k* and their communities of each node when optimizing 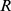 and 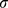.

	*R*				*S*		
Name	*T_R_*_,*i*_	Community	Δ*k*	Name	*T_σ_*_,*i*_	Community	Δ*k*
VPc	50	Auditory	4	5Bm	43	Somato-motor	−6
2	50	Somato-motor	7	20b	40	Visual	0
AMLS	48	Visual	7	AII	39	Auditory	1
21b	48	Visual	4	AI	37	Auditory	−1
PS	48	Visual	7	PS	36	Visual	7
21a	47	Visual	5	5Am	36	Somato-motor	−8
ALLS	45	Visual	4	PMLS	35	Visual	2
Sb	45	Frontolimbic	8	VPc	34	Auditory	4
Hipp	45	Frontolimbic	2	61	34	Somato-motor	0
AAF	44	Auditory	3	3a	33	Somato-motor	2
Tem	42	Auditory	2	6 m	33	Somato-motor	−4
P	40	Auditory	3	Cga	33	Frontolimbic	−13
SIV	40	Somato-motor	5	7	32	Visual	−1
3a	39	Somato-motor	2	Tem	32	Auditory	2
1	39	Somato-motor	5	1	32	Somato-motor	5
DLS	38	Visual	1	20a	31	Visual	−6
SII	38	Somato-motor	3	PFCI	31	Frontolimbic	−10
PSb	38	Frontolimbic	3	ALLS	30	Visual	4
4	36	Somato-motor	3	SII	30	Somato-motor	3
PLLS	33	Visual	5	SSAi	30	Somato-motor	−5
AII	33	Auditory	1	PFCMiI	30	Frontolimbic	−3
RS	32	Frontolimbic	−2	CGp	30	Frontolimbic	−10
PMLS	30	Visual	2	36	30	Frontolimbic	9
20b	30	Visual	0	5AI	29	Somato-motor	−10
VLS	29	Visual	−2	5BI	28	Somato-motor	−10
PFCMiI	29	Frontolimbic	−3	3b	27	Somato-motor	1
Enr	29	Frontolimbic	−1	4 g	27	Somato-motor	−1
19	27	Visual	3	2	26	Somato-motor	7
3b	27	Somato-motor	1	4	26	Somato-motor	3
17	25	Visual	1	21b	25	Visual	4
SSAo	25	Somato-motor	−5	SIV	25	Somato-motor	5
18	22	Visual	2	Ia	25	Frontolimbic	−3
4 g	21	Somato-motor	−1	Ig	25	Frontolimbic	5
AI	20	Auditory	−1	AES	24	Visual	−1
PFCI	20	Frontolimbic	−10	AAF	24	Auditory	3
36	20	Frontolimbic	9	P	24	Auditory	3
61	19	Somato-motor	0	19	23	Visual	3
7	18	Visual	−1	RS	23	Frontolimbic	−2
SSAi	16	Somato-motor	−5	Enr	23	Frontolimbic	−1
5Bm	14	Somato-motor	−6	21a	22	Visual	5
Ig	14	Frontolimbic	5	35	22	Frontolimbic	7
6 m	12	Somato-motor	−4	17	21	Visual	1
PFCMd	11	Frontolimbic	−6	VLS	21	Visual	−2
AES	10	Visual	−1	EPp	21	Auditory	−6
Ia	9	Frontolimbic	−3	AMLS	20	Visual	7
EPp	8	Auditory	−6	PFCMd	20	Frontolimbic	−6
5BI	7	Somato-motor	−10	18	19	Visual	2
Cga	6	Frontolimbic	−13	DLS	18	Visual	1
35	5	Frontolimbic	7	SSAo	18	Somato-motor	−5
5Am	4	Somato-motor	−8	Sb	17	Frontolimbic	8
20a	3	Visual	−6	PLLS	13	Visual	5
CGp	2	Frontolimbic	−10	PSb	13	Frontolimbic	3
5AI	1	Somato-motor	−10	Hipp	11	Frontolimbic	2

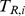
 and 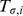 can be seen from Eqs. (3) and (4), respectively.

### Controlling Communities of the Cortical Network - a Mesoscopic Way

In the following, we show which module/community is significant to be controlled in a mesoscopic way. According to Table 2, we sort and choose the nodes with 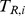 and 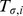 in the first 
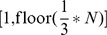
 as controlling nodes (CN), 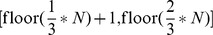 as intermediate controlling nodes (ICN) and 
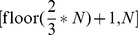
 as weak controlling nodes (WCN), respectively. The number of CN, ICN and WCN in each community are presented in Tables 3 and 4. We also calculate the proportions of the locations of CN, ICN and WCN in each community, respectively. Tables 3 and 4 show that most of the areas in the community Auditory serve as CN. Specifically, when minimizing 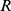, most of the areas in the community Visual work as CN and ICN, most of the areas in the community Somato-motor belong to ICN and WCN and most of the areas in the community Fronto-limbic serve as ICN and WCN. When minimizing 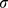, most of the areas in the community Visual work as ICN and WCN, most of the areas in the community Somato-motor belong to CN and ICN and most of the areas in the community Fronto-limbic serve as WCN. From the above observations, when minimizing 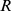, the importance of each community is listed in a descending order: 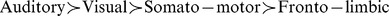. When minimizing 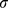, the importance of each community is listed in a descending order: 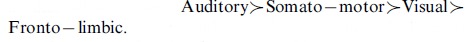 Hence, although the community Auditory is sparsely connected with other communities and is the smallest community, it is the most important one to control the cortical network. The observed phenomenon indicates that community with sparse connection to other communities should be paid special attention to control the network efficiently.

**Table 3:** The proportions of controlling nodes in four communities when minimizing 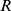.

	Number of CN	Number of ICN	Number of WCN	percent for CN	percent for ICN	percent for WCN
Visual	6	7	3	6/16 = 37.5%	7/16 = 43.75%	3/16 = 18.75%
Auditory	4	2	1	4/7 = 57.1%	2/7 = 28.57%	1/7 = 14.29%
Somato-motor	5	4	7	5/16 = 31.25%	4/16 = 25%	7/16 = 43.75%
Fronto-limbic	2	5	7	3/14 = 14.29%	5/14 = 35.71%	7/14 = 50%

**Table 4:** The proportions of controlling nodes in four communities when minimizing 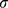.

	Number of CN	Number of ICN	Number of WCN	percent for CN	percent for ICN	percent for WCN
Visual	5	3	8	5/16 = 31.25%	3/16 = 18.75%	8/16 = 50%
Auditory	4	1	2	4/7 = 57.1%	2/7 = 14.29%	1/7 = 28.57%
Somato-motor	6	9	1	6/16 = 37.5%	9/16 = 56.25%	1/16 = 6.25%
Fronto-limbic	2	5	7	2/14 = 14.29%	5/14 = 35.71%	7/14 = 50%

## Discussion

The cortical hubs are believed to play pivotal roles in the coordination of information processing in cortical networks. In previous studies, the identification and classification of hub regions have been analyzed in terms of node degree, structural motif, path length, clustering coefficient distributions and synchronization [20], [22]. In these works, the intrinsic relationship between structural and functional connectivity is analyzed by using ensembles of neurons coupled by a cortical network of cats’ brain. By means of statistical methods, the crucial importance of nodes and clusters are revealed to analyze the separation and integration of sensory information in the cerebral cat cortex [24], [46].

Additionally, one of the major challenges for human is to control natural systems or networks efficiently. As a typical natural network, identifying controlling nodes of a realistic anatomical network of cat cortical connectivity is of crucial significance to provide insights into avoiding abnormal synchronization in typical neural diseases [8], [9], [12]. In the light of previous studies, the problem of identification of controlling nodes of cortical networks remains open.

In this study, we have investigated the identification of controlling nodes in a network representing the connectivity among cortical areas in cats’ brain. The issue regarding controllability of the cortical network is converted into a combinatorial optimization problem [43]. A representative evolutionary computation method, JaDE, which is a self-adaptive and efficient algorithm to solve real-world optimization problems [38], is used to identify controlling nodes with an appropriate encoding scheme. The comparison with some well-known network-based methods and evolutionary computation methods is presented, revealing JaDE performs best among all the algorithms.

The controlling nodes of the cortical network are detected in microscopic, mesoscopic and macroscopic ways. Using such various scales will help us to understand the controllability of neuronal networks in depth. We have shown a close relationship of the number of driver nodes and the locations of the driver nodes, indicating a concave shape of the mean degree of driver nodes as an increase of the number of driver nodes. For low values of the number of driver nodes, the areas with a large degree govern the coordination dynamics of the network. As a whole, the nodes with a small degree are important to be selected as controlling regions, which is in contrast to the work in [22] and supports the finding in [29], [32]. More importantly, the most prominent community in the cortical network of cats is the community Auditory, which has sparse connections with other communities. The comparative results of two quantities for measuring controllability of complex networks are also investigated in detail.

The model and methods can be extended and improved in several ways. Firstly, it is meaningful to propose more efficient optimization methods to deal with controllability of cortical networks. Secondly, we have only focused on the highest level of cortical networks and thus large subnetworks [14], [47], [48] with other biologically realistic features [11], [49], [50] should be considered. Finally, the results should be applied to other realistic natural systems to illustrate controlling rules. The achievements would require further developments in neuroscience, in the theory of dynamical complex networks, in optimization methods as well as in control science.

## Materials and Methods

### Notations

Throughout this paper, 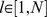 denotes the number of driver nodes of a network. 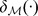 denotes the characteristic function of the set 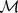, i.e., 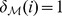 if 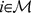; otherwise, 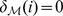. Define a graph by 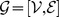, where 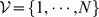 denotes the vertex set and 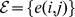 the edge set.

### Cortico-cortical Network of Cats’ Brain

The cortico-cortical network of cats’ brain is a biological network that describes the anatomical connectivity of cats’ brain [18], [19]. Here, we use a version of a dataset in [21]. The cat cerebral cortex can be divided into 53 cortical areas, linked by about 830 fibres of different densities into a weighted and directed complex network. It consists of four topological clusters that broadly agree with four functional cortical sub-divisions: visual cortex (16 areas), auditory (7 areas), somato-motor (16 areas) and fronto-limbic (14 areas). We also refer to the topological clusters as communities or modules. The community Auditory is sparsely connected while the communities Visual, Somato-Motor and Fronto-Limbic are densely connected among each other [16].

### Model and Problem Formulation

We consider a reference evolution/state as follows:


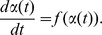


This equation is general, since many real-world systems such as social networks, biological systems and other natural systems can be modeled as differential equations [30].

Then, the following model of a diffusively coupled array of identical systems is considered as a general complex network:


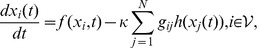
(5)

where 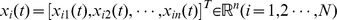 is the state vector of the 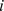 th node and 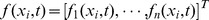 is a continuous vector function. 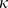 is the coupling gain of the network. In the coupling term, the node is connected through a generic output function 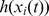. The matrix 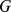 stands for the connectivity about the cortical network topology. The graph 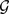 is supposed to be directed, weighted and simple (without self-loops and multiple edges). Let weighted and directed matrix 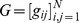 be the adjacency matrix of graph 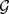, which is defined as follows: for any pair 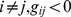 if 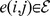; otherwise, 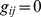. 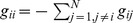 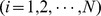. The adjacency matrix 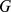 can be converted into the Laplacian matrix 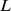 by neglecting the weights over the networks. For any pair 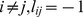 if 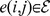; otherwise, 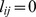. 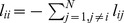, 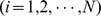. The output degree 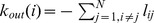 of a node 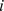 is the number of efferent connections that it projects to other nodes, and its input degree 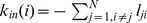, is the number of the afferent connections it receives. Denote by 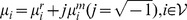, the set of eigenvalues of 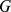 and assume that they are ordered in such a way that 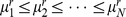.

To control such a cortical network to the reference evolution 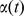, feedback controllers are added to (5):


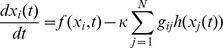



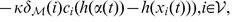
(6)

where 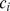 are control gains or coupling strengths. Suppose that 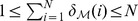. We aim to lead the cortical network (5) toward the desired reference evolution 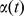, i. e., 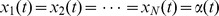.

By linear manipulations, the stability analysis of (6) can be transformed into the dynamics of 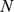 independent blocks in the parameters 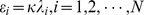 [26], [51], [52],


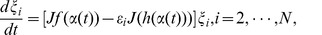
(7)

where 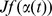 and 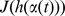 are the Jacobians of the functions 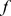 and 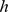 calculated around the time varying reference evolution 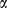. 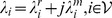 are the eigenvalues of the 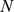-dimensional structural matrix


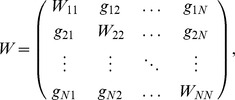


where 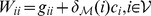. Without loss of generality, we assume that 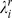 are sorted as 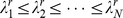.

As pointed out in [25], [26], through above transformation, the problem of controllability of complex networks is converted into synchronizability of networks. Similar to the analysis method of checking synchronizability of networks, the enhancement of controllability can be characterized by reducing the eigenratio.


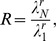


and make


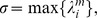


as small as possible [25], [44], i. e., the smaller the 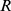 and 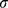 are, the easier the network is controllable. Previous works have shown that 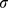 can be neglected, since usually 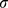 is very small and has only minor effects on synchronizability/controllability of networks [45]. We also consider 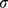 and illustrate the impact of 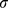 on controllability, since 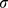 is important when one considers some special graphs, e. g., normalized Laplacian graph.

It should be noted that the selection of driver nodes is a typical combinatorial optimization problem [43], where the locations of driver nodes are discrete variables, and the design of control gains is a continuous optimization problem. Taking the locations of driver nodes and their control gains into account together, the controllability of networks can be viewed as a multimodal optimization problem.

Here, minimizing 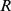 and 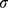 by determining locations of driver nodes 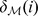 and designing 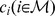 can be formulated as follows:


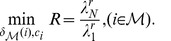
(8)


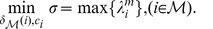
(9)

From the above equations, we study the controllability of cortical networks by minimizing 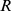 and 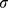, respectively. Evolutionary computation methods are employed to study the controllability and identify controlling regions.

### The Strategies for Determining the Locations of Driver Nodes

Several well-known strategies for determining the locations of driver nodes or controlling nodes are illustrated as follows [43].

1. **Degree-based strategies**. Degree-based pinning schemes are the most popular methods to select potential driver nodes, in which the locations of driver nodes are chosen according to degree information of networks in a decreasing or an ascending way [25], [30], [53]. Here, the two schemes are called ascending and descending degree-based strategies, respectively. The output degree 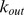 is used to provide degree information.
2. **Betweenness centrality (BC)-based strategies**. Similar with the degree-based scheme, we consider descending and ascending BC-based strategies.
3. **Closeness-based strategies**. Two kinds of closeness-based strategies, i. e. descending and ascending closeness-based strategies are taken into account.
4. **Node importance-based strategies**. Since the controllability of the cortical network is mainly related to its eigenvalues, it is interesting to determine the locations of driver nodes by considering their importance in the network [54]. We analyze two measures of node importance for the cortical network. The first one is to minimize 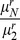 of 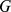 upon sequential removal of nodes, which is called 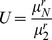-**based strategy**. The other one is to minimize 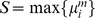 of 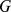 upon sequential removal of nodes, which is called 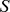-**based strategy**. It should be noted that 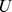 and 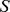 are usually used to measure synchronizability performance of complex networks [44].
5. **Evolutionary algorithm-based strategies**. Using an appropriate encoding scheme, differential evolution (DE) is used to select driver nodes and design control gains. Evolutionary algorithms have been successfully used in the synchronization of two coupled systems in [55], the coordination of unmanned aircraft vehicle [43] and networks topology with optimal synchronizability [56]. Here, adaptive differential evolution is adopted to identify the controlling nodes [38].

In the degree-based, the BC-based, the closeness-based and the node importance-based strategies, control gains in all the nodes are considered to be identical and one can tune the control gains of driver nodes in the cortical network gradually with a step size 0.1, like [25], [26].

### Differential Evolution and its Encoding Scheme

In order to determine the locations of driver nodes in the cortical network and design their control gains, an appropriate encoding scheme is used according to [43]. In addition, equipped with this encoding scheme, JaDE [38] is used to detect the controlling nodes/areas/regions of the cortical network of cats’ brain in microscopic, mesoscopic and macroscopic ways, respectively.
