# Correction: Identifying Controlling Nodes in Neuronal Networks in Different Scales

**DOI:** 10.1371/annotation/08d4312c-cce3-4975-8b3d-e7978857ffa4

**Published:** 2012-08-07

**Authors:** Yang Tang, Huijun Gao, Wei Zou, Jürgen Kurths

There was an error in the affiliations of Jürgen Kurths. The correct affiliations are:

2 Institute of Physics, Humboldt University Berlin, Berlin, Germany,

3 Potsdam Institute for Climate Impact Research, Telegraphenberg, Potsdam, Germany,

5 Institute for Complex systems and Mathematical Biology, University of Aberdeen, Aberdeen, United Kindom 

